# Racial Identity-Rooted Academic Motivation of First-Year African American Students Majoring in STEM at an HBCU

**DOI:** 10.3389/fpsyg.2021.669407

**Published:** 2021-06-08

**Authors:** Kimberley Edelin Freeman, Cynthia Eileen Winston-Proctor, Felicia Gangloff-Bailey, Jason M. Jones

**Affiliations:** ^1^Department of Human Development & Psychoeducational Studies, Howard University, Washington, DC, United States; ^2^School of Education, Graduate School of Arts & Sciences, Howard University, Washington, DC, United States; ^3^Department of Psychology, Howard University, Washington, DC, United States; ^4^College of Arts & Sciences, Graduate School of Arts & Sciences, Howard University, Washington, DC, United States; ^5^SOL Development, Oakland, CA, United States; ^6^Department of Psychological Sciences, Winston-Salem State University, Winston-Salem, NC, United States

**Keywords:** academic motivation, HBCUs, open-ended survey question, African Americans, undergraduate students, STEM

## Abstract

The purpose the present study is to explore African American undergraduate students' perceptions of their experiences and academic motivation within a Historically Black College or University (HBCU) learning environment. As part of a larger study, we collected 212 open-ended survey responses from first year students in STEM majors about how the HBCU context shapes their academic motivation. We used semantic thematic data analysis and found three major themes and corresponding sub themes that were salient in the development of students' academic motivation: place (institutional climate, HBCU mission and tradition, and absence of marginalization); pedagogy (culturally relevant pedagogy, positive faculty-student relationships, African American curriculum and instruction, racial socialization); and people (people “like me”; student, faculty and alumni models of high achieving African Americans). We discovered that HBCU institutional factors engendered academic motivation that is rooted in students' racial identity and suggest the construct of racial identity-rooted academic motivation. Given the important and unique realities of African American students that impact their educational experiences, engagement, identity development, and achievement in various types of school contexts, self and sociocultural variables must be included in research and theory on the motivational psychology of African American students. Implications for higher education practice and future research are discussed.

The psychological science of broadening participation can offer needed insights into factors that promote the achievement of African American students in science, technology, engineering, and mathematics (STEM) fields. Academic motivation, for example, is a key variable in students' academic achievement. Yet, traditional academic motivation research and theory have not adequately represented the motivational and educational attributes of African American students. Despite the study of academic motivation of African American students having a long history within the field of educational psychology, cultural integrity approaches are lacking in the extant research and this has limited the validity and utility of the knowledge base. Most motivation theories and research are based in whiteness (Usher, 2018). There is also a lack of deep knowledge about the development of African American students' academic motivation in varying educational contexts. For decades, scholars, such as Banks et al. (1978), Cokley (2001, 2015), Freeman et al. (2002), and Graham and Hudley (2005), have called for more diverse research approaches and theories in the study of the motivational psychology of African American students. In order to better understand the motivation of African American students and provide research evidence that can be used to improve practice, sociocultural variables must be included in research given the important and unique history and realities of African American students that impact their educational experiences, engagement and achievement (Banks et al., 1978; Cokley, 2003; Graham and Hudley, 2005; DeCuir-Gunby and Schutz, 2014). The present study uses an open-ended survey to explore the views of first-year undergraduate students in STEM majors at an Historically Black University about how the Black educational context shapes their academic motivation.

For more than a century, Historically Black Colleges and Universities (HBCUs) have played a significant role in the provision of higher education to African Americans and others. Founded to provide higher education to African Americans who could not attain it otherwise in the system of segregated schooling, HBCUs have enrolled and produced more than their share of African American college students and graduates. Although HBCUs make up <3% of all colleges and universities, they award more than 15% of all bachelor's degrees to African Americans each year (National Science Foundation, 2016). In addition, in STEM fields, year after year several HBCUs make the list of top producers of African American undergraduates who go onto earn Ph.D.'s in STEM (National Science Foundation, 2016). Thus, studying the education of HBCUs can offer new insight into broadening participation of African American students in STEM. Yet, HBCUs are rarely the subject of study within educational psychology research on the motivation and achievement of African American students. While research that compares the motivation of white and African American students typically depicts African American students' school motivation and achievement as lacking or low, HBCU research on HBCU students reveals positive academic and psychological development of African American students. These unique Black educational contexts are critical socializers of many African American students and studying students in HBCU contexts can provide more culturally grounded research regarding the academic motivation of African American students. Thus, examining HBCU students and contexts in depth is warranted.

The goal of this study is to uncover students' perceptions of their first-year college experiences and academic motivation within an HBCU learning environment. In addition, the study seeks to contribute to the developing psychological science of broadening participation in STEM by describing how HBCUs influence the academic motivation of first-year African American students majoring in STEM. More specifically, we study students at HBCUs to capture and understand African American students' motivation from a culturally-grounded and cultural integrity approach (Burrell et al., 2013; Lee et al., 2019). Cultural integrity approaches focus on the centrality of culture in learning and development, and the strengths and cultural resources that diverse learners bring to the educational context (Boykin, 2000). In addition, in contrast to a common focus in educational psychology on understanding African American students' motivation in relation to experiencing racial stigma, threatening conditions and hostile environments, it is important to examine motivation in racially-affirming contexts, such as HBCUs. Examining the motivation patterns of students at HBCUs can provide new insight into the motivational psychology of African American students.

## Review of Related Literature

### Research on African American Students' Motivation and Achievement at HBCUs

There is a large body of scholarship that provides evidence of the effectiveness of HBCUs in terms of: graduating black students; graduates' labor market outcomes; providing greater access to higher education for first generation college students and low-income students than other colleges and universities; advancing social justice, Black heritage and community development; supportive campuses; and producing undergraduates that have higher graduate school participation rates than African American undergraduates from non-HBCUs (Constantine, 1994; Wenglinsky, 1996; Nettles et al., 1999; Gasman et al., 2010; Price et al., 2011; Arroyo and Gasman, 2014). In addition, compared to African American students attending non-HBCUs, African American students at HBCUs report more faculty-student interactions, more positive relationships with faculty, more faculty encouragement and support, and greater participation in research experiences with faculty (Cokley, 2003, 2005; Kim and Conrad, 2006; Gasman et al., 2010; Hurtado et al., 2011; Fountaine, 2012). African American students attending HBCUs also report that they grow more in terms of personal and academic development in college than African American students attending non-HBCUs (Nelson Laird et al., 2007; Sadler, 2014).

Biographies and testimonies of HBCU alumni describe the motivating nature and impact of HBCUs, such as making students feel confident, driven, competent, valued, supported, and expected to achieve. Cokley (2000) examined predictors of academic self-concept among African American students attending HBCUs compared to African American students attending Predominately White Institutions (PWIs). Cokley found that for students at HBCUs, quality of faculty-student relationships and GPA were both strong, positive predictors of academic self-concept, but for students at PWIs, GPA was a much stronger positive predictor of self-concept than quality of student-faculty relationships, which was also a significant positive predictor. Cokley replicated this study in 2002 with a larger sample and found similar but not identical results. In this sample, GPA was the only significant predictor of academic self-concept for African American students attending PWIs, but for African American students attending HBCUs quality of faculty-student relationships was the stronger predictor, followed by GPA. For the most part, studies show that African American students attending HBCUs experience greater intellectual gains, and more positive self-concept and personal development than African American students attending predominately white institutions (Freeman and Thomas, 2002).

### Studies of Motivation of African American Undergraduate Students in STEM at HBCUs

Ireland (2016) examined the motivation of 77 Black undergraduate women in computing majors and found that women enrolled at HBCUs had lower psychological cost, lower identity interference, and higher intention to remain in computing majors than women attending non-HBCUs. In a survey study of 639 African American undergraduate women in engineering majors, Rockliffe (2020) found that women attending HBCUs (*n* = 131) had significantly higher engineering identity, academic self-efficacy, and fewer incidents of racial microaggressions than African American women in engineering majors at non-HBCUs. Lopez et al. (2006) administered a survey to 1,789 undergraduate students in computer science (CS) majors at 21 HBCUs and 21 non-HBCUs. They found that HBCU CS students had lower mathematics self-efficacy than CS students at PWIs. Also, women had lower computer self-efficacy than men among White CS students, but not among African American CS students. Lent et al.'s (2005) survey study of 487 undergraduate students in engineering majors at two HBCUs and one PWI showed that the HBCU students reported significantly higher academic self-efficacy, outcome expectations, technical interests, social supports, and goals than students at PWIs. Smith et al.'s (2014) study of 14 HBCU students in engineering majors showed how social responsibility and community were part of students' motivation to achieve in engineering. Finally, White et al. (2019) gave a survey to 374 students from five HBCUs and found that science efficacy, racial identity and science grades were positively related.

The extant research, which consists mostly of between-group comparative, survey studies, shows a more positive psychological profile of African American students attending HBCUs than African American students attending non-HBCUs, but they provide little detailed and contextualized evidence about the nature of HBCU learning environments and how motivation is cultivated in these contexts. Social variables, such as positive relationships with faculty and peers, and self-beliefs, such as self-efficacy and self-concept, are key in students' motivation and achievement in STEM fields (Museus et al., 2011). But most studies look at differences between students' characteristics and outcomes at HBCUs and non-HBCUs, and rarely examine what is occurring in the institutions that is contributing to differences in students' attributes. Although HBCUs are known as top producers of Blacks in STEM, their institutional contexts are not sufficiently studied to gain evidence about dimensions of the HBCU context and education that promote positive student development and outcomes. Understanding multiple institutional factors that explain the success of HBCUs in enhancing students' motivation and achievement in STEM is needed, however research on institutional factors that account for the large number of Black college graduates in STEM is limited (Museus et al., 2011).

There are a few studies, with small numbers of schools and participants, that have examined the institutional characteristics and practices of HBCUs that are related to students' motivation and achievement. Freeman et al. (2008) conducted a quasi-experimental study at two HBCUs and found that participating in collaborative learning communities in STEM classes was positively related to students' intrinsic motivation. In a case study of African American women in STEM majors at a Historically Black College for women, Perna et al. (2009) found that the HBCU provided an institutional climate that promoted students' psychological readiness to be successful in STEM including a cooperative peer culture, faculty accessibility, and undergraduate research opportunities. In a qualitative case study of six African American women who graduated from HBCUs for their undergraduate degree and went on to graduate programs in mathematics or chemistry at PWIs, Joseph (2012) found that students described that HBCUs nurtured their self-concept and prepared them to be successful in graduate school environments through hands-on faculty and a strong sense of identity. These studies reveal aspects of institutional climate and faculty practice that enhance students' motivation and achievement in STEM. More studies that use open-ended and qualitative methods to examine how the HBCU context shapes students' motivation and achievement, across a range of HBCUs, are warranted. In addition, research that examines how HBCU institutional characteristics interact with students' motivation variables is needed (Gasman et al., 2010).

### The Present Study

The purpose of this study is to explore how HBCUs shape the academic motivation of first-year students in STEM majors. We use a distinctive multimethod approach to capture these students' own words to describe their educational experiences. In so doing, we adopt a pragmatic theoretical perspective, as well as survey research design methodology that targets a single open-ended response prompt and a thematic analysis method to interpret students' first-year experiences in an HBCU learning environment that are related to their academic motivation. The open-ended survey responses provide “natural language” typical in qualitative inquiry in psychology (see Levitt et al., 2018), as well as compelling and conceptually complicated vantage points from which to explore students' motivational psychology. Furthermore, in organizational research, “open-ended questions are used … to explore, explain, and/or reconfirm existing ideas” (Jackson and Trochim, 2002, p. 308). Geer (1991) also provides evidence that open-ended survey questions can be used to measure salient topics with undergraduate students. Finally, our position as researchers is insiders of HBCUs (the first two authors are HBCU faculty; and all authors are HBCU graduates) and thus we have extensive varied experience at HBCUs and interpret the data with this knowledge base.

## Method

### Participants and Data Sources

The present study is part of a 4-year (2008–2012) longitudinal, concurrent mixed-methods study funded by the National Science Foundation Historically Black Colleges and Universities Undergraduate Program (HBCU-UP) Education Research Project^1^ grant program. The objective of the larger study was to examine what works in producing African American science and mathematics teachers at HBCUs. One component of the project included a 4-year longitudinal quantitative survey of a freshmen cohort of STEM majors at a flagship, private HBCU. The sample for the survey was recruited from the 2008–09 freshmen cohort of STEM majors attending college for the first time. In total, 323 students were included in the sample, which represented ~80% of the total freshmen STEM majors at the university that year. The baseline sample was 71% women, 65% biology majors, 18% engineering majors, 7% chemistry majors, 4% computer science majors, and 2% mathematics majors. More than 80% of the sample identified as Black/African Americans (81%) and 14% identified as Multiple Race including Black. The average level of education among students' mothers and fathers was some college.

### Procedures

Students were recruited during freshman week at the university, which was before classes began. Researchers recruited students in dormitories, orientation meetings, and other events. We conducted several in-person group survey administrations, to collect multiple baseline measures from the students during freshman week. After the baseline survey, all surveys were administered to the students via the web, using the SurveyMonkey online survey tool. Written informed consent was obtained from all participants before they completed each survey. Participants received $10 for completing the baseline survey, and $15 for each subsequent survey. The data for the present study come from the survey that was administered online to the students at the end of their first semester. On the survey, students were asked to explain their perceived importance of attending an HBCU for their academic motivation using the following open-ended question “what is the value of attending an HBCU in terms of your educational ambitions?” We phrased the question in this manner to use neutral language that would elicit responses that focused on students' motivation and the impact of attending a Black college on students' motivation. In total, we collected 212 open-ended responses.

### Data Analytic Approach

Data analysis was conducted by three researchers and occurred in several stages. We conducted semantic thematic analysis in order to uncover patterns in the data about students' motivation characteristics and how HBCUs shape students' academic motivation. We used an inductive approach with open coding as our goal was to develop codes from the raw data and not use a priori codes. We coded each open-ended response and through analysis and interpretation linked codes into categories and larger themes. According to Braun and Clarke (2006), “a theme captures something important about the data in relation to the research question, and represents some level of patterned response or meaning with the data set” (p. 10). We followed the procedures for semantic thematic data analysis as described by Braun and Clarke (2006), which included the following steps: (1) reading and re-reading the data to become familiar with it; (2) generating initial codes; (3) searching for themes; (4) reviewing themes; and (5) defining and naming themes.

In the first stage of analysis, all three researchers read through of all the data and developed codes and a codebook. We then coded a portion of the responses together and revised the codes and codebook for clarity, removal of redundant codes, etc. In the next step, two of the three researchers coded all the data by hand and then entered all of the coded data into the QSR *NVivo 9* software for further analysis. We used to *NVivo* to conduct code frequency analysis and code relational analysis. We used the results of the code frequency and relational analyses to augment our interpretation of how codes related to each other and combined into themes, which facilitated our process of searching for themes, and defining and naming themes and sub-themes. Throughout data analysis, each researcher wrote “analytic memos” to record the process of coding, interpretations during analysis, and development of themes (Miles et al., 2014). We engaged in regular team meetings to discuss the ongoing analysis and emergent themes and revised themes to ensure that each theme was accurately reflected by the data and codes, and that themes (and their sub-themes) were distinct from each other. Upon triangulation and consensus across all three researchers, final themes and sub-themes were decided.

## Results

Three themes emerged regarding the salient dimensions of HBCUs that influenced students' motivation: Place: The HBCU Context; Pedagogy: Culturally Relevant Pedagogy; and People: The Impact of being Surrounded by High Achieving African Americans (people “like me”). As shown in Table 1, each theme had several sub-themes. Place included positive institutional climate, HBCU mission and tradition, and absence of marginalization in school. Culturally relevant pedagogy, faculty-student relationships, curriculum, instruction, and racial socialization were components of pedagogy. People referred to faculty, students, and alumni at HBCUs that constitute a large group/ concentration of models (people “like me”) who are exemplars of Black educational achievement. Taken together, these themes reflect the ways that students describe how attending an HBCU positively influences their academic motivation. Each theme is described in greater detail below with corresponding illustrative student quotes.

**Table 1:** Themes and subthemes in how the HBCU Shapes Students' Academic Motivation.

**Category/Sub-themes**	**Theme**
Positive Institutional Climate HBCU Mission and Tradition Absence of Marginalization in School	Place: The HBCU Context
Faculty Caring and Faculty-Student Relationships African-American Curriculum and Instruction Racial Socialization	Pedagogy: Culturally Relevant Pedagogy
Like Me Models	People: The impact of being surrounded by high-achieving African Americans (people “like me”)

### Place: The HBCU Context

#### HBCU Mission and Tradition

Founded to educate freed African American slaves, HBCUs have been providing educational opportunity and higher education to African American (and other) students for more than 150 years. The mission and tradition of HBCUs resonate today on campuses as much as they did at the founding of HBCUs in the 19th century, which was expressed by students as important for their academic motivation in responses, such as the following: “There is so much history at [the HBCU]. Many greats have walked the paths of [the HBCU], and the legacy lives on through their descendants.” (Qt1) And,

I feel that the pride that is also foster in the African American community and Black heritage is also greatly due to the high esteem that HBCUs were founded upon and those values in turn foster a certain drive in black students. (Qt2)

The greatest value of attending a historically black university is the connection to history you feel. When I arrived at [HBCU] and visited the museum in [the] library I saw a picture from 1950 of women standing in the building I now live in**;** I felt as if I was a part of history. That feeling encourages me to do the best I can. (Qt3)

The HBCU in this study has a strong academic reputation and track record of producing high-achieving African American graduates, particularly in STEM. The physical environment captures this legacy and embodies the mission and tradition of African American achievement. The place represents achievement, and you feel it when you are in that place, which shapes students' motivation. The history of achievements permeates the physical environment and institutional climate.

#### Positive Institutional Climate

HBCUs are widely known for having an institutional climate that is supportive, nurturing, and family-like. We also found ample evidence of this value of HBCUs in the present study. The relatedness dimension of the climate—nurturing, belongingness, and positive relationships—was repeatedly cited as a major value of HBCUs. Supportive institutional climate was a significant influence on students' achievement motivation, as shown in responses, such as these: “I feel that the greatest value of attending a historically black university is the close knit family environment that the campus provides” (Qt4); “I feel that I will experience a special kind of nurturance that will prepare me for a successful career” (Qt5); and

…. I know for me as a student, so I started in HBCU and then I went to [PWI] and then I went to [another PWI], but I needed to be at a HBCU. I don't think that all HBCU's are equal [but] one thing that is unique to a HBCU is that it is nurturing. (Qt6)

Positive relationships in particular are important features of the HBCU context and climate that enhance students' motivation. Students describe that they need this type of nurturing environment to promote their success.

#### Absence of Marginalization in School

Another important dimension of the climate included an educational environment in which students were not marginalized because of their race, and a place that provides a counternarrative to the prevailing stereotypes and negative narratives about African Americans that are pervasive in broader society. Many students contrasted experiences in predominately white institutions and HBCUs. Some talked about the positive influence of the HBCU environment in contrast to the all-white school environments they had attended previously. The following responses describe this nature of the HBCU context:

Firstly it was important to me to remove myself from the all white environment I was previously in, for the fact that I believed that I was at a disadvantage compared to my other non-black classmates. [HBCU] is a non-bias school where I can be judged on the quality of my work. (Qt7)

… the HBCU experience is unlike any other. As a Black student who has been educated in predominately white schools for the majority of my education, I have experienced the struggle of attempting to receive an education as a minority; while I went to the top schools in my state and was successful in my studies, my achievements were always secondary to those of my white counterparts. I was marginalized in my educational societies, and only received academic and social recognition from teachers and peers when I far exceeded the school's expectations. On the converse, at [HBCU] I have felt the remarkable power of being educated amongst my social and cultural peers. Our race ties us together because of our shared common history; I could not receive this educational experience at any PWI. For many Black students, the chance to live and learn amongst a racial majority establishes a sense of equality and self-worth that was previously unexplored. (Qt8)

I think that a lot of people are convinced that you can't be successful and be “black” at the same time. Attending a historically black university introduces you to the range of people included within the range, all with their minds set on success. It can change your perception and increase your confidence in yourself and your race. (Qt9)

Students describe how being in the HBCU space allows them to see Black success, which refutes prevailing stereotypes about African Americans, makes them proud, raises their confidence and expectancies for success, and is motivating. At HBCUs, racial marginalization, stereotypes, and master narratives about the intellectual inferiority of African Americans are shattered. One aspect of culturally relevant pedagogy is disrupting inequities in education and economic opportunities for African Americans (Ladson-Billings, 2013; Leonard and Martin, 2013), which is also the HBCU mission.

### Pedagogy: Culturally Relevant Pedagogy

Pedagogical practices of HBCU faculty were a salient contributor the development of motivation of first year students. Faculty approaches and instructional practices that students described can be viewed as culturally relevant pedagogy (Ladson-Billings, 1995; Howard and Rodriguez-Minkoff, 2017). Culturally relevant pedagogy is teaching that uses students' culture as a vehicle and asset for learning in the classroom, fosters high achievement and cultural identity among African American students, and promotes critical consciousness among teachers and students. The culturally relevant pedagogy included several subthemes: faculty caring and faculty-student relationships; African American focused curriculum and instruction; and racial socialization.

#### Faculty Caring and Faculty-Student Relationships

Students favorably described the caring and commitment of faculty, positive relationships among faculty and students, and faculty high expectations for student success. The following responses are two of the many student responses that highlighted the instrumental role of faculty in the development of students' motivation and achievement:

The greatest value of attending a historically black university is the professors. The professors in the … department really want to help each student do the best they can while at [college] and once they leave. They go the extra mile by looking over your applications to programs or talking to you about career choices. I don't think students receive that level of care at other universities. (Qt10)
…I plan on going into the medical profession and I feel that by attending a HBCU it has better prepared me to go on and succeed in medical school, much more than a state school or non-HBCU would. I believe an HBCU provides a more academically encouraging environment in which the faculty care about your success. Personally, for me, I do not believe I would be as involved academically with working alongside faculty doing research if I was at any other institution. (Qt11)

Students discussed faculty caring and positive relationships as critical influences on their motivation. Students explained how faculty are dedicated to their success, supportive and encouraging and this enhances students' engagement and positive emotions. Students describe a connection with faculty and feeling like faculty understand and identify with them. Some students describe HBCUs as being different from non-HBCUs in terms of the level of faculty nurturing, student-centeredness, caring about the well-being and development of the students, and commitment to ensuring students' success. Students identify and feel a relatedness with faculty, and this enhances their motivation in school, as said by this student: “I think your professors identify with the students more at HBCU's. It's comforting and encouraging.” (Qt12) HBCU faculty are described as hands on and having high expectations for the academic success of African American students, which has been shown in other studies (Gasman et al., 2010; Joseph, 2012).

#### African-American Curriculum and Instruction

In addition, students emphasized the effectiveness of faculty teaching and the motivating aspects of course content, which included a focus on African American history, culture and accomplishments. Students made the following types of responses that reflect this sub-theme: “Professors at HBCUs understand how we as blacks learn and can relate to us,” (Qt13) and,

[HBCU] is really exposing me to a lot of things historically, professionally, and socially that I do not feel I could have learned at a non-HBCU. It teaches me the things about the African American race and history that I was not taught in school making me more confident and proud to be a black woman. (Qt14)

I think the one of the best aspects of attending an HBCU is the well-rounded education you receive. Each class I have taken at [HBCU] teaches things outside of the Western ideas or perspectives, and also incorporates black history and culture into the lessons. (Qt15)

HBCUs are well-known reservoirs of Black culture and history; many aspects of the HBCU curricula incorporate African-American, African, and Black studies and history, providing their students with a strong sense of their history, culture, and purpose. As repositories for black heritage, HBCUs are rich arenas that help promote and incorporate Black and African history in American society and more importantly, my life. (Qt16)

Students described faculty using teaching methods that build in and on students' background knowledge, interests, and cultural characteristics, and they use material that is relevant to students. Students learned new knowledge about African Americans and other multicultural content and this engaged and motivated students and enhanced their sense of self-efficacy and identity. As one student described the education at the HBCU: “…you get a sense of culture as well as a good education.” (Qt17) Because students are gaining new knowledge about African Americans and their history and accomplishments, this enhances students' regard for African Americans, or racial pride in the achievements of African Americans, and this enhances students' motivation.

#### Racial Socialization

Faculty also engage in racial socialization of students, preparing them for careers as well as the world—as Black people. Racial socialization is interactions between teachers and students about what is means to be Black in America and how to thrive in a racialized context (Stevenson et al., 2002). Above and beyond teaching academic subjects, faculty engage actively in socializing students in terms of how to prepare for future careers as African Americans, as two students described: “Teachers give you a perspective of what it will be like in America's workforce as an African American.” (Qt18) And, “Going to a HBCU gets you ready to move to the next level because it properly prepares you for the things you will encounter in leaving a black university going to a white university.” (Qt19)

Racial socialization is a part of culturally relevant teaching for African American undergraduate students, because work and graduate education are highly racialized and racial socialization can positively prepare students for those next levels given that racial socialization is positively associated with school and mental health outcomes for African Americans (Nebblett et al., 2012). Foster (2008) describes how graduate schools and companies recruit HBCU students because of the unique academic preparation and psychological strengths they gained at HBCUs. The data show that culturally relevant pedagogy can have a significant positive influence on the racial identity, self-efficacy and academic motivation of African American students.

### People: The Impact of Being Surrounded by High-Achieving African Americans (People “Like Me”)

Repeatedly throughout the responses, students expressed that interacting, working and learning with similar others (i.e., African Americans who are attaining academic success) is confidence-building and motivating. Numerous times in the data, students described the powerful and positive effect of being surrounded by people “like me.” The following statement, “Being surrounded by people who look like me and are focused, intelligent, and driven toward a positive goal is amazing. It makes me proud and pushes me to do better” (Qt20), and the statements below are some of the many responses that reflect this theme:

When I was in Canada, I never had a teacher who was like me. It wasn't until I came to the States and then it impressed me to no end that I had black teachers teaching math and science. And, I just felt like, wow, you know, had I not come so far, I wouldn't, I would never have known that we even existed and that we were capable of doing this, and doing it well. (Qt21)

Every day we are surrounded by amazing people. Whether it happens to be a professor or a student, we are all striving for excellency. Instead of walking around and seeing people that don't look like me, I'm reminded how beautiful the black culture is and what positive things we are capable of. (Qt22)

The greatest value of attending an HBCU is seeing other black people working hard to become professionals in so many different fields. This makes me more confident in myself and makes me more motivated to make an effort to excel academically. (Qt23)

The responses demonstrate how models boost self-efficacy, racial pride/racial identity, and motivation for African American students, which is aligned with the principles of social cognitive theory (Bandura, 1986). Bandura explains that “successes by others raises observers' outcome expectations and judgments of their own performance capabilities” (p. 301). And, “… it is probably the more proximal successes of similar peers that operate as recurring motivators in day-to-day activities” (p. 302). Observing similar others achieve boosts one's sense of self-efficacy. In addition, the responses reflect how the students' motivation is rooted partly in their racial identity. Being surrounded by other African Americans who are attaining success and who they identity with, has a powerful positive impact on their own academic motivation.

## Discussion

The purpose of the present study was to use open-ended survey data to explore how HBCUs shape African American first-year STEM students' academic motivation. In so doing, we used students' natural language to explain “the value of attending an HBCU for their ambitions.” As such, the study provides an initial understanding of the sociocultural and situational nature of academic motivation afforded by an HBCU learning context. First-year STEM students described experiences within their college learning environment that included the following: (1) being taught Black and multicultural content in their courses by faculty who care about them and want them to excel; (2) being surrounded by and experiencing relatedness with people like them who are models of African American education and career success; and (3) the nurturing nature of the HBCU context and climate. In addition, this study illustrates the utility of using open ended survey data as a rich source of insight (Miller and Lambert, 2014) into complex phenomena. It also supports Gergen et al.'s (2015) assertion about the promises of the use of qualitative data to advance cultural understanding.

The findings highlight the social-cognitive nature of African American students' motivational psychology and the importance of context, culture, models and social relationships (Eccles and Wigfield, 2020). Bandura (1977, 1986) suggests that most human learning occurs observationally, through modeling. In this study, models were salient and provide a representation of where identity and motivation are linked for African American students: they observe peers, faculty and alumni in the HBCU context and they identity with these models of success, which shapes their motivational psychology. Faculty-student relationships and faculty caring are essential parts of an HBCU culturally responsive education and the cultivation of students' motivation and achievement. As described by Usher (2018), “The teacher-student relationship is a daily occasion for the affirmation (or denial) of one's personhood” (p. 141). Social variables are significant sources of African American students' academic self-efficacy, which has been found in other studies (Cokley, 2000, 2002). Relatedness, vicarious experiences, and social learning are primary sources of HBCU students' motivation and achievement.

In addition, the findings underscore that social, emotional, and psychological well-being are foundational to students' successful learning experiences. The HBCU educational environment is designed intentionally to focus on personal and academic development of African American students, and the context creates racial and personal pride and other positive emotions (e.g., joy), which promote students' social and emotional development. Social-emotional learning (SEL) supports students' positive identity development, self-efficacy, and motivation in school (CASEL, 2020; McKinney de Royston et al., 2020). Positive social relationships in school are particularly important for academic success of African American students, which emanates from the shared cultural values of communalism and cultural socialization in the African American community (Boykin, 1986; Hilliard et al., 1990; Hilliard, 1995). In addition, the African American tradition in education and African-centered education, focus on the holistic development of African American students including agency, cultural identity, intellectual, spiritual, and moral development (Lee, 2008; Shockley and Lomotey, 2020), which also embodies the principles of SEL. In sum, good schools support students' holistic development, identities, and social-emotional development (McKinney de Royston et al., 2020).

### Conceptual Framework: Racial Identity-Rooted Academic Motivation

Furthermore, it stood out to us that the students' responses reflect how HBCU institutional factors engender academic motivation that is rooted partly in students' racial identity. That is, from their experiences in the HBCU context, students have a boost in their racial identity, such as racial pride and racial private regard (Sellers et al., 1997), and this becomes a source of students' academic motivation—leading to what we call racial identity-rooted academic motivation. Being surrounded by and engaging with other African Americans who are attaining success, and who they identity with, boosts students' racial identity, and self-efficacy, and has a significant impact on their academic motivation. In another study that was part of our larger project (Freeman et al., 2012), we found that in the first year of college the racial identity (private regard) of this group of students increased significantly (Mean at baseline = 4.12, Mean at end of year 1 = 5.90). In addition, we found that students that had larger increases in private regard had higher grades (3.5 cumulative GPA and above) than students with smaller increases in private regard (<3.5 cumulative GPA). Our findings are consistent with other studies that show that high private regard (racial pride) is associated positively with motivation and academic achievement for African American students (Chavous et al., 2003; Griffin et al., 2012; Hope et al., 2013).

**Figure 1:**
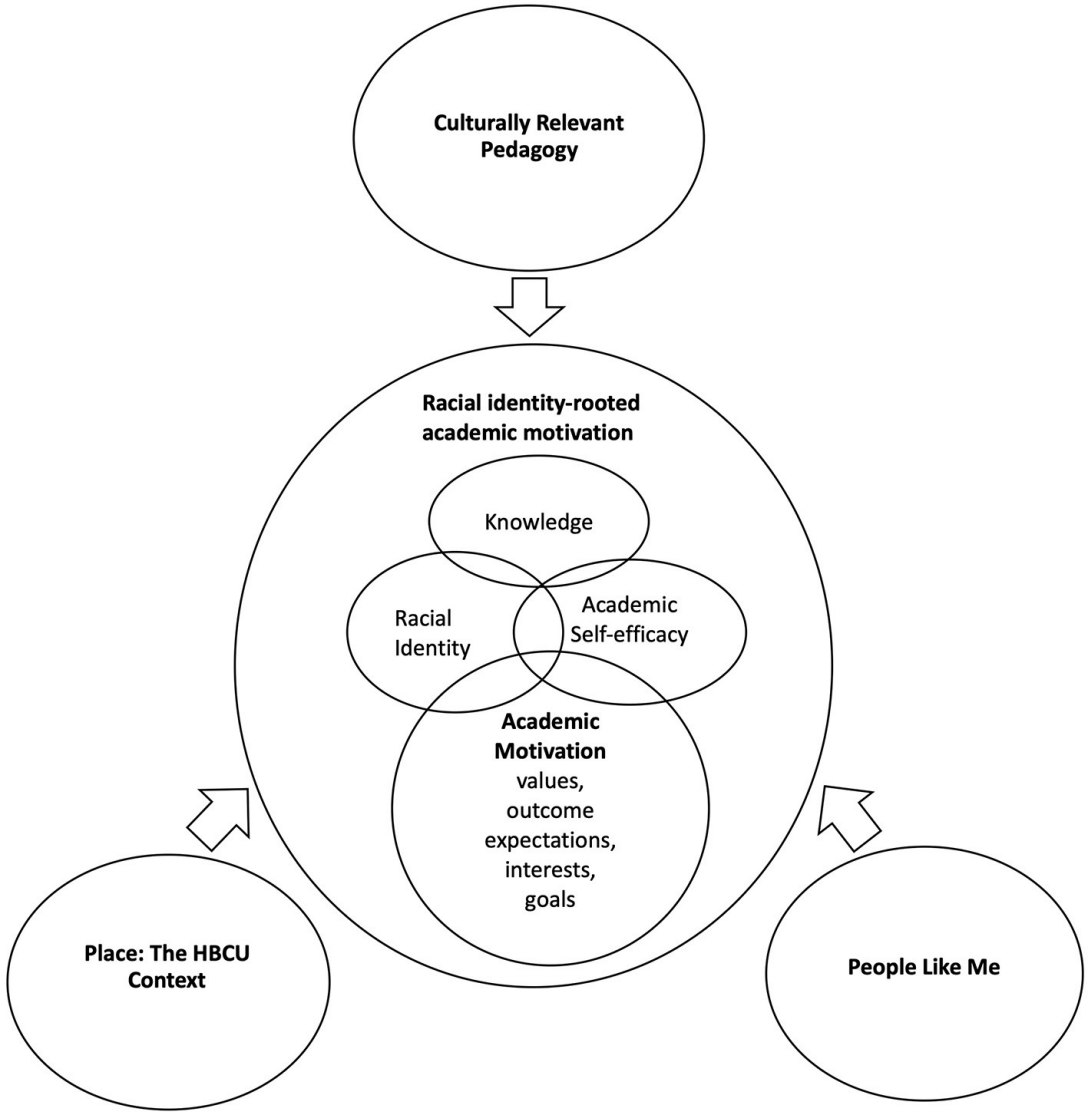
Conceptual framework: The development of racial identity-rooted academic motivation at an HBCU.

In Figure 1 we propose a conceptual model of how dimensions of the HBCU context cultivate African American students' academic motivation, through self-efficacy and racial identity. When African American students have a positive experience within the HBCU context (place), and with people and pedagogy, their knowledge, racial pride and identity, and self-efficacy are enhanced. The physical, psychological, intellectual and social experience of the HBCU campus, shapes students' knowledge and self-beliefs, particularly racial identity (private regard) and self-efficacy, which promote students' academic motivation (outcome expectations, interests, values and goals). Statements, such as the following represent racial identity-rooted academic motivation: “Attending a historically black university…can change your perception and increase your confidence in yourself and your race.” “Being surrounded by people who look like me and are focused, intelligent, and driven toward a positive goal is amazing. It makes me proud and pushes me to do better.” Students gain new knowledge in their classes through culturally relevant pedagogy, and racial identity, self-efficacy and academic motivation are sprung up: “It teaches me the things about the African American race and history that I was not taught in school making me more confident and proud to be a black woman.” Students' experiences in the HBCU context significantly impact their sense of self, which in turn increases their motivation; for example as reflected in this statement from a student who saw a picture from 1950 of women in her dorm: “I felt as if I was a part of history. That feeling encourages me to do the best I can.” The student's motivation to “do the best I can” is fostered by the connection/identity she felt with the HBCU/others in the HBCU community. Self-efficacy and racial identity are positively shaped by the HBCU context and are significant positive predictors and components of academic motivation for African American students. The construct of racial identity-rooted academic motivation aligns conceptually with the Eccles (2009) expectancy-value theory model connecting social identities to motivation, and Oyserman's (2015) identity-based motivation theory.

To summarize, the study illustrates how dimensions of the HBCU context enhance African American first year students' knowledge and self-beliefs, particularly racial private regard and self-efficacy, which promote students' racial identity-rooted academic motivation. The findings depict ways that HBCUs provide person-context congruence for African American students. In HBCU contexts, African American students feel cultural and racial context congruence, which fosters school belonging and the positive development of students' identity and motivation (Byrd and Chavous, 2011). Jackson (2013) found similar results in a study of 12 Black women majoring in STEM who transferred to a Southern HBCU from community colleges. The students describe the HBCU as a safe place to develop an identity in STEM. In contrast to most K-12 schools and historically white institutions, HBCUs provide African American students an experience of academic achievement being the rule of the Black experience and not the exception or something that Black students have to choose to do at the expense of their racial identity as has been documented in other studies (e.g., Rodgers, 2008), and this is motivating.

### Implications for Practice

Institutions of higher education can use the results of this study to inform their own practice aimed at Black college student success (Arroyo and Gasman, 2014). The natural language and themes generated in this study provide real examples of culturally relevant teaching that can be understood by others and used by others in their practice. Culturally relevant pedagogy included students' engagement with diverse literature and perspectives in courses, including and especially regarding African American achievements and history. It also included racial socialization in the form of teaching and messages about being Black in America and how to be successful being Black in America. Historically White Institutions can re-do their curricula to remove racist and marginalizing content and add more diverse content. In addition, the findings highlight that faculty are key in the training the next generation of scientific talent (Hurtado et al., 2011). Recruiting new diverse faculty with different worldviews and critical consciousness should be a priority for institutions. Faculty development in the areas of culturally relevant and anti-racist education, and teaching to promote college student identity, motivation and socioemotional development are important areas for strategic planning and investment also. Additionally, providing spaces for African American students to create community, strong relationships and cultural experiences like the HBCU experiences described in this study can be an institutional goal.

### Limitations and Future Research

One limitation of the present study is the results may be generalizable only to African American students in STEM majors at HBCUs. Additionally, given that women made up 71% of the participants, findings may be limited to women at HBCUs. We did not explore the findings for men and women separately which may have masked some important gender differences and intersectional experiences. For example, Cokley (2001), using a sample of 258 undergraduate HBCU students, found that women reported higher levels of academic motivation than men, and also racial identity, academic self-concept, and intrinsic motivation were positively correlated for women, but they were not among men. Gendered and intersectional experiences of HBCU students, in STEM fields and more broadly, need further study. Also, compared to in-depth qualitative methods, the single open-ended survey question may be limited in its ability to capture students' experiences in the HBCU context and the situated and sociocultural nature of academic motivation. Future qualitative research using interview and observation methods can examine more deeply the social, observational, and culturally sustaining learning that occurs in HBCUs.

Finally, we encourage future race-focused educational psychology research to examine culturally affirming experiences and strengths-based approaches to African American students' academic motivation, to add new types of sociocultural knowledge to the knowledge base that mostly focuses on understanding the impact of negative experiences, such as racism and marginalization. Research on racism and racial barriers that African American students face is important. Some African American students are motivated to attain school success as a form of resistance to racism (Nasir et al., 2016). Yet, it is just as important to examine the meaning and significance of African and African American historical legacy, achievement, culture and community for African American students' motivation, strivings, and how they navigate schooling. This is an avenue for future research.

## Conclusion

We use a rare approach to capture students' own words to describe their educational experiences. The open-ended survey responses provided “natural language” typical in qualitative inquiry in psychology (see Levitt et al., 2017, 2018), as well as compelling and conceptually complicated vantage points from which to explore students' motivational psychology. Our position as researchers is insiders of HBCUs and we have extensive varied experience at HBCUs. Taken together, reporting all of these elements of our study and discussing what they might suggest for advancing knowledge in educational psychology about HBCUs illustrates a form of methodological integrity that is aligned with the new American Psychological Association (APA) Journal Article Reporting Standards (JARS) (American Psychological Association, 2020).

Given the important and unique realities of African American students that impact their educational experiences, engagement, identity development, and achievement in various types of school contexts, self and sociocultural variables must be included in research on the motivational psychology of African American students (Graham, 1994; Graham and Hudley, 2005). In addition, the freshman year experience is critical in determining college success, especially in the gateway STEM courses. Studying African American college students in an HBCU learning context is a key point of differentiation in this study from others in educational psychology and within the science of broadening participation (see Ireland et al., 2018). During late adolescence and early adulthood, identity development is salient, and colleges play a key socializing role in nurturing positive identities and academic motivation of students. African American college students' identity development is not only complicated by their entry into the developmental stage of emerging adulthood, but also by the psychological complexity of the meaning of race within the culture of a racialized society (Winston et al., 2004; Winston, 2012; Winston and Winston, 2012). This study offers new culturally-specific knowledge about self and school context factors that are important to understand the motivational psychology of African American students. HBCUs create a learning environment in which students during the first year of college can see themselves in ways that are in stark contrast to broader social roles defining what African Americans ought to be like. The developmental timing of this early college experience is potentially transformative because young adulthood is a critical time in students' personal and academic development.

## Data Availability Statement

The original contributions presented in the study are included in the article/supplementary material, further inquiries can be directed to the corresponding author/s.

## Ethics Statement

The studies involving human participants were reviewed and approved by the Howard University Institutional Review Board. The patients/participants provided their written informed consent to participate in this study.

## Author Contributions

KF contributed to the study design, data collection, data analysis, and writing the article. CW-P contributed to the study design, data collection, and writing the article. FG-B and JJ contributed to the data analysis and writing the article. All authors contributed to the article and approved the submitted version.

## Conflict of Interest

The authors declare that the research was conducted in the absence of any commercial or financial relationships that could be construed as a potential conflict of interest.

## Footnotes

^1^This program is now called the HBCU-UP Broadening Participation Research (BPR) Projects.

## References

[B1] American Psychological Association (2020). Publication Manual of the American Psychological Association, 7th Edn. Washington, DC: American Psychological Association.

[B2] Arroyo A. T. Gasman M. (2014). An HBCU-based educational approach for black college student success: toward a framework with implications for all institutions. Am. J. Educ. 121, 57–85. 10.1086/678112

[B3] Bandura A. (1977). Toward a unifying theory of behavioral change. Psychol. Rev. 84, 191–215. 10.1037/0033-295X.84.2.191 847061

[B4] Bandura A. (1986). Social Foundations of Thought and Action: A Social Cognitive Theory. Upper Saddle River, NJ: Prentice Hall.

[B5] Banks C. McQuater G. Hubbard J. (1978). Toward a reconceptualization of the social-cognitive bases of achievement orientations in Blacks. Rev. Educ. Res. 48, 381–397. 10.3102/00346543048003381

[B6] Boykin A. W. (1986). “The triple quandary and the schooling of Afro-American children,” in The School Achievement of Minority Children: New Perspectives, ed Neisser U. (Hillsdale, MI: Lawrence Erlbaum Associates, Publishers), 57–92.

[B7] Boykin A. W. (2000). The talent development model of schooling: placing students at promise for academic success. J. Educ. Stud. Placed Risk 5, 3–25. 10.1207/s15327671espr0501&2_2

[B8] Braun V. Clarke V. (2006). Using thematic analysis in psychology. Qual. Res. Psychol. 3, 77–101. 10.1191/1478088706qp063oa

[B9] Burrell J. O. Winston C. E. Freeman K. E. (2013). Race acting: the varied and complex affirmative meaning of “acting Black” for African American middle school students in a mathematics and science charter school. Cult. Psychol. 19, 95–116. 10.1177/1354067X12464981

[B10] Byrd C. M. Chavous T. (2011). Racial identity, school racial climate, and school intrinsic motivation among African American youth: the importance of person–context congruence. J. Res. Adolesc. 21, 849–860. 10.1111/j.1532-7795.2011.00743.x

[B11] CASEL (2020). CASEL's SEL Framework: What Are the Core Competence Areas and Where Are They Promoted? Available online at: https://casel.org/wp-content/uploads/2020/12/CASEL-SEL-Framework-11.2020.pdf (accessed April 21, 2021).

[B12] Chavous T. M. Bernat D. H. Schmeelk-Cone K. Caldwell C. H. Kohn-Wood L. Zimmerman M. A. (2003). Racial identity and academic attainment among African American adolescents. Child Dev. 74, 1076–1090. 10.1111/1467-8624.00593 12938705

[B13] Cokley K. (2000). An investigation of academic self-concept and its relationship to academic achievement in African American college students. J. Black Psychol. 26, 148–164. 10.1177/0095798400026002002

[B14] Cokley K. (2001). Gender differences among African American students in the impact of racial identity on academic psychosocial development. J. Coll. Stud. Dev. 42, 480–487. Available online at: https://www.researchgate.net/publication/2326019346

[B15] Cokley K. (2002). The impact of college racial composition on African American students' academic self-concept: a replication and extension. J. Negro Educ. 71, 288–296. 10.2307/3211181

[B16] Cokley K. (2003). What do we know about the motivation of African American students?: Challenging the “anti-intellectual” myth. Harvard Educ. Rev. 73, 524–558. 10.17763/haer.73.4.3618644850123376

[B17] Cokley K. (2005). Racial(ized) identity, ethnic identity, and afrocentric values: conceptual and methodological challenges in understanding African American identity. J. Counsel. Psychol. 52, 517–526. 10.1037/0022-0167.52.4.517

[B18] Cokley K. (2015). The Myth of Black Anti-Intellectualism: A True Psychology of African American Students. Santa Barbara, CA: Praeger.

[B19] Constantine J. (1994). The “added value” of historically black colleges. Academe 80, 12–17. 10.2307/40250608

[B20] DeCuir-Gunby J. T. Schutz P. A. (2014). Researching race within educational psychology contexts. Educ. Psychol. 49, 244–260. 10.1080/00461520.2014.957828

[B21] Eccles J. (2009). Who am I and what am I going to do with my life? Personal and collective identities as motivators of action. Educ. Psychol. 44, 78–89. 10.1080/00461520902832368

[B22] Eccles J. S. Wigfield A. (2020). From expectancy-value theory to situated expectancy-value theory: a developmental, social cognitive, and sociocultural perspective on motivation. Contemp. Educ. Psychol. 61:101859. 10.1016/j.cedpsych.2020.101859

[B23] Foster L. (2008). “Foreword,” in Ebony Towers in Higher Education: The Evolution, Mission, and Presidency of Historically Black Colleges and Universities, eds Brown R. Brown C. (Sterling, TX: Stylus), ix–xiii.

[B24] Fountaine T. P. (2012). The impact of faculty-student interaction on Black doctoral students attending historically black institutions. J. Negro Educ. 81, 136–147. 10.7709/jnegroeducation.81.2.0136

[B25] Freeman K. Thomas G. E. (2002). Black colleges and college choice: characteristics of students who choose HBCUs. Rev. High. Educ. 25, 349–358. 10.1353/rhe.2002.0011

[B26] Freeman K. E. Alston S. Winborne D. G. (2008). Do learning communities enhance the quality of students' motivation and learning in STEM? J. Negro Educ. 77, 227–240. Available online at: https://www.jstor.org/stable/25608689

[B27] Freeman K. E. Gutman L. M. Midgley C. (2002). “Can achievement goal theory enhance our understanding of the achievement motivation and performance of African American young adolescents?” in Goals, Goal Structures, and Patterns of Adaptive Learning, ed Midgley C. (Mahwah, NJ: Lawrence Erlbaum Associates.), 175–204

[B28] Freeman K. E. Winston C. E. Anderson A. (2012). ““Use-Inspired Research” on the psychology of success in STEM at an HBCU: Racial identity, motivation and achievement trajectories,” in Paper Presented at the Annual Meeting of the American Educational Research Association (Vancouver, CA).

[B29] Gasman M. Lundy-Wagner V. Ransom T. Bowman N. (2010). Unearthing promise and potential: our nation's historically black colleges and universities. ASHE High. Educ. Rep. 35, 1–134. 10.1002/aehe.v35:5

[B30] Geer J. G. (1991). Do open-ended questions measure ‘salient' issues? Public Opin. Q. 55, 360–370. 10.1086/269268

[B31] Gergen K. J. Josselson R. Freeman M. (2015). The promises of qualitative inquiry. Am. Psychol. 70, 1–9. 10.1037/a0038597 25581004

[B32] Graham S. (1994). Motivation in African Americans. Rev. Educ. Res. 64, 55–117. 10.3102/00346543064001055

[B33] Graham S. Hudley C. (2005). “Race and ethnicity in the study of motivation and competence,” in Handbook of Competence and Motivation, eds Elliot A. Dweck C. (New York, NY: The Guilford Press), 392–413.

[B34] Griffin T. M. Chavous T. Cogburn C. Branch L Sellers R. (2012). Dimensions of academic contingencies among African American college students. J. Black Psychol. 38, 201–227. 10.1177/0095798411414892

[B35] Hilliard A. G. (1995). The Maroon Within Us: Selected Essays on African American Community Socialization. Baltimore, MD: Black Classic Press.

[B36] Hilliard A. G. Payton-Stewart L. Williams L. O. (Eds.) (1990). “Infusion of African and African American content in the school curriculum,” in Proceedings of the First National Conference (Chicago: Third World Press).

[B37] Hope E. C. Chavous T. M. Jagers R. J. Sellers R. M. (2013). Connecting self-esteem and achievement: diversity in academic identification and dis-identification patterns among Black college students. Am. Educ. Res. J. 50, 1122–1151. 10.3102/0002831213500333

[B38] Howard T. Rodriguez-Minkoff A. (2017). Culturally relevant pedagogy 20 years later: Progress or pontificating? What have we learned, and where do we go? Teach. Coll. Rec. 119, 1–32. Available online at: https://www.tcrecord.org

[B39] Hurtado S. Eagan K. M. Tran M. C. Newman C. B. Chang M. J. Velasco P. (2011). “We do science here”: underrepresented students' interactions with faculty in different college contexts. J. Soc. Issues 67, 553–579. 10.1111/j.1540-4560.2011.01714.x 23503924 PMC3596161

[B40] Ireland D. T. (2016). The roles of social identities in the achievement motivation and retention of black undergraduate women in computing disciplines (Doctoral dissertation), Howard University, Washington, DC, United States.

[B41] Ireland D. T. Freeman K. E. Winston-Proctor C. DeLaine K. McDonald-Lowe S. Woodson K. (2018). Unhidden figures: a synthesis of research examining the intersectional experiences of black women and girls in STEM education. Rev. Res. Educ. 42, 226–254. 10.3102/0091732X18759072

[B42] Jackson D. L. (2013). A balancing act: impacting and initiating the success of African American female community college transfer students in STEM into the HBCU environment. J. Negro Educ. 82, 255–271. 10.7709/jnegroeducation.82.3.0255

[B43] Jackson K. M. Trochim W. M. (2002). Concept mapping as an alternative approach for the analysis of open-ended survey responses. Organ. Res. Methods 5, 307–336. 10.1177/109442802237114

[B44] Joseph J. (2012). From one culture to another: years one and two of graduate school for African American women in the STEM fields. Int. J. Doctor. Stud. 7, 125–142. 10.28945/1571

[B45] Kim M. M. Conrad C. F. (2006). The impact of historically black colleges and universities on the academic success of African-American students. Res. High. Educ. 47, 399–427. 10.1007/s11162-005-9001-4

[B46] Ladson-Billings G. (1995). Toward a theory of culturally relevant pedagogy. Am. Educ. Res. J. 32, 465–491. 10.3102/00028312032003465

[B47] Ladson-Billings G. (2013). “Stakes is high”: educating new century students. J. Negro Educ. 82, 105–110. 10.7709/jnegroeducation.82.2.0105

[B48] Lee C. (2008). Synthesis of research on the role of culture in learning among African American youth: the contributions of Asa G. Hilliard, III. Rev. Educ. Res. 78, 797–827. 10.3102/0034654308320967

[B49] Lee J. Y. Khalil D. Boykin A. W. (2019). Enhancing STEM teaching and learning at HBCUs: a focus on student learning. New Direct. Stud. Serv. 167, 23–36. 10.1002/ss.20318

[B50] Lent R. W. Brown S. D. Sheu H. Schmidt J. Brenner B. R. Gloster C. S.. (2005). Social cognitive predictors of academic interests and goals in engineering: utility for women and students at historically black universities. J. Counsel. Psychol. 52, 84–92. 10.1037/0022-0167.52.1.84

[B51] Leonard J. Martin D. (2013). The Brilliance of Black Children in Mathematics: Beyond the Numbers and Toward New Discourse. Charlotte, NC: Information Age Publishing.

[B52] Levitt H. M. Bamberg M. Creswell J. W. Frost D. Josselson R. Suárez-Orozco C. (2018). Journal article reporting standards for qualitative research in psychology: the APA Publications and Communications Board Task Force report. Am. Psychol. 73, 26–46. 10.1037/amp0000151 29345485

[B53] Levitt H. M. Motulsky S. L. Wertz F. J. Morrow S. L. Ponterotto J. G. (2017). Recommendations for designing and reviewing qualitative research in psychology. Qual. Psychol. 4, 2–22. 10.1037/qup0000082

[B54] Lopez A. M. Jr. Giguette M. S. Schulte L. J. (2006). “Large dataset offers view of math and computer self-efficacy among computer science undergraduates,” in ACM-SE 44: Proceedings of the 44th Annual Southeast Regional Conference (Melbourne, VIC), 10–12. 10.1145/1185448.1185484

[B55] McKinney de Royston M. Lee C. Nasir N. Pea R. (2020). Rethinking schools, rethinking learning. Phi Delta Kappan 102, 8–13. 10.1177/0031721720970693

[B56] Miles M. B. Huberman A. M. Saldana J. (2014). Qualitative Data Analysis: A Methods Sourcebook, 3rd Edn. Thousand Oaks, CA: Sage Publications, Inc.

[B57] Miller A. L. Lambert A. D. (2014). Open-ended survey questions: item nonresponse nightmare or qualitative data dream? Surv. Pract. 7, 1–14. 10.29115/SP-2014-002426451335

[B58] Museus S. D. Palmer R. T. Davis R. J. Maramba D. C. (2011). Racial and ethnic minority students' success in STEM education. ASHE High. Educ. Rep. 36, 53–85. 10.1002/aehe.v36.6

[B59] Nasir N. S. Rowley S. J. Perez W. (2016). “Cultural, racial/ethnic, and linguistic diversity and identity,” in Handbook of Educational Psychology, 3rd Edn., eds Corno L. Anderman E. (New York, NY: Routledge), 186–195.

[B60] National Science Foundation (2016). Special Report NSF 19–304: Women, Minorities, and Persons with Disabilities in Science and Engineering. U.S. Department of Education. Available online at: https://ncses.nsf.gov/pubs/nsf19304/data

[B61] Nebblett E. W. Rivas-Drake D. Umana-Taylor A. J. (2012). The promise of racial and ethnic protective factors in promoting ethnic minority youth development. Child Dev. Perspect. 6, 295–303. 10.1111/j.1750-8606.2012.00239.x

[B62] Nelson Laird T. Bridges B. Morelon-Quainoo C. Williams J. Holmes M. (2007). African American and Hispanic student engagement at minority serving and predominantly white institutions. J. Coll. Stud. Dev. 48, 39–56. 10.1353/csd.2007.0005

[B63] Nettles M. T. Perna L. W. Freeman K. E. (1999). Two Decades of Progress: African Americans Moving Forward in Higher Education. Falls Church, VA: Frederick D. Patterson Research Institute.

[B64] Oyserman D. (2015). “Identity-based motivation,” in Emerging Trends in the Social and Behavioral Sciences, eds Scott R. Kosslyn S. (Hoboken, NJ: John Wiley & Sons, Inc.), 1–11. 10.1002/9781118900772.etrds0171

[B65] Perna L. Lundy-Wagner V. Drezner N. Gasman M. Yoon S. Bose E.. (2009). The contributions of HBCUs to the preparation of African American women for STEM careers: a case study. Res. High. Educ. 50, 1–23. 10.1007/s11162-008-9110-y

[B66] Price G. N. Spriggs W. Swinton O. (2011). The relative returns of graduating from a historically black college/university: propensity score matching estimates from the national survey of Black Americans. Rev. Black Polit. Econ. 38, 103–130. 10.1007/s12114-011-9088-0

[B67] Rockliffe F. (2020). Examining interrelationships among gender and racial microaggressions, self-efficacy, interests, outcome expectations, engineering identity, and persistence goals for black undergraduate women (Doctoral dissertation), Howard University, Washington, DC, United States.

[B68] Rodgers K. A. (2008). Racial identity, centrality and giftedness: expectancy-value application of motivation in gifted African American students. Roeper Rev. 30, 111–120. 10.1080/02783190801955103

[B69] Sadler C. (2014). A multi-level mixed methods study of institutional context, climate, competence, and relatedness on black college students' gains (Doctoral dissertation), Howard University, Washington, DC, United States.

[B70] Sellers R. M. Rowley S. A. J. Chavous T. M. Shelton J. N. Smith M. A. (1997). Multidimensional inventory of Black identity: a preliminary investigation of reliability and construct validity. J. Pers. Soc. Psychol. 73, 805–815. 10.1037/0022-3514.73.4.805

[B71] Shockley K. G. Lomotey K. (Eds.) (2020). African-Centered Education: Theory and Practice. Gorham, ME: Myers Education Press, LLC.

[B72] Smith K. C. Fleming L. N. Moore I. N. Burris S. E. Bornmann F. (2014). “African American undergraduate success in engineering: the “prove them wrong” syndrome or social responsibility,” in Paper Presented at 2014 ASEE Annual Conference (Indianapolis, IN). Available online at: https://peer.asee.org/20036

[B73] Stevenson H. C. Cameron R. Herrero-Taylor T. Davis G. Y. (2002). Development of the teenager experience of racial socialization scale: correlates of race related socialization frequency from the perspective of Black youth. J. Black Psychol. 28, 84–106. 10.1177/0095798402028002002

[B74] Usher E. (2018). Acknowledging the whiteness of motivation research: seeking cultural relevance. Educ. Psychol. 53, 131–144. 10.1080/00461520.2018.1442220

[B75] Wenglinsky H. H. (1996). The educational justification of historically Black colleges and universities: a policy response to the U.S. Supreme Court. Educ. Eval. Policy Anal. 18, 91–103. 10.3102/01623737018001091

[B76] White A. M. DeCuir-Gunby J. Kim S. (2019). A mixed methods exploration of the relationships between the racial identity, science identity, science self-efficacy, and science achievement of African American students at HBCUs. Contemp. Educ. Psychol. 57, 54–71. 10.1016/j.cedpsych.2018.11.006

[B77] Winston C. E. (2012). “Human personality: race self complexity and symbolic meaning of persons living race in American society and culture,” in Cultural Psychology of Human Values, eds Branco A. U. Valsiner J. (Charlotte, NC: Information Age Publishing), 163–194.

[B78] Winston C. E. Rice D. W. Bradshaw B. Lloyd D. Harris L. Burford T.. (2004). “Race self complexity, science success and narrative theories of personality: how is race represented in the self and identity construction of African American adolescents?” in The Interplay Between Self and Social Process in Science and Math Achievement: New Directions in Child and Adolescent Development, eds Bouchey H. Winston C. E. (New York, NY: Jossey-Bass), 55–77. 10.1002/cd.116 15707162

[B79] Winston C. E. Winston M. R. (2012). “Cultural psychology of racial ideology in historical perspective: an analytic approach to understanding racialized societies and their psychological effects on lives,” in Oxford Handbook of Culture and Psychology, ed Valsiner J. (New York, NY: Oxford University Press), 559–581. 10.1093/oxfordhb/9780195396430.013.0026

